# Mutation tendency of mutator *Plasmodium berghei* with proofreading-deficient DNA polymerase δ

**DOI:** 10.1038/srep36971

**Published:** 2016-11-15

**Authors:** Hajime Honma, Mamoru Niikura, Fumie Kobayashi, Toshihiro Horii, Toshihiro Mita, Hiroyoshi Endo, Makoto Hirai

**Affiliations:** 1 Department of International Affairs and Tropical Medicine, Tokyo Women’s Medical University, 8-1 Kawada-cho, Shinjuku, Tokyo, Japan; 2 Department of Infectious Diseases, Kyorin University School of Medicine, 20-2, Shinkawa 6, Mitaka City, Tokyo, Japan; 3 Department of Molecular Protozoology, Research Institute for Microbial Diseases, Osaka University, 3-1 Suita, Osaka, Japan; 4 Department of Molecular and Cellular Parasitology, Juntendo University, 2-1-1 Hongo, Bunkyo, Tokyo, Japan

## Abstract

In this study, we investigated the mutation tendency of a mutator rodent malaria parasite, *Plasmodium berghei*, with proofreading-deficient DNA polymerase δ. Wild-type and mutator parasites were maintained in mice for over 24 weeks, and the genome-wide accumulated mutations were determined by high-throughput sequencing. The mutator *P. berghei* had a significant preference for C/G to A/T substitutions; thus, its genome had a trend towards a higher AT content. The mutation rate was influenced by the sequence context, and mutations were markedly elevated at TCT. Some genes mutated repeatedly in replicate passage lines. In particular, knockout mutations of the AP2-G gene were frequent, which conferred strong growth advantages on parasites during the blood stage but at the cost of losing the ability to form gametocytes. This is the first report to demonstrate a biased mutation tendency in malaria parasites, and its results help to promote our basic understanding of *Plasmodium* genetics.

Malaria is caused by infection by members of the genus *Plasmodium*, and during 2013, it was responsible for 197 million malaria cases and 584 thousand deaths^1^. Malaria is still a serious public health concern in tropical and subtropical areas. Treatment for malaria mainly comprises chemotherapy with antimalarial drugs, but the most virulent malaria parasite *Plasmodium falciparum* has developed resistance to almost all conventional drugs^2^. Artemisinin-based combination therapies are currently recommended as first-line treatments by the World Health Organization, but parasites resistant to artemisinins have been reported recently^3^,^4^. These issues make malaria control more complex and demand the development of a new approach to control malaria based on advanced knowledge of the parasites. The recent popularisation of next-generation sequencing (NGS) techniques has made the genomes of several *Plasmodium* species available^5^,^6^,^7^,^8^,^9^,^10^, and novel genetic tools have also been developed^11^,^12^,^13^,^14^,^15^. These developments have motivated the determination of previously unknown functions of genes to accelerate our understanding of the biology of these parasites. However, most of the existing tools are restricted to applications based on reverse genetics. The *piggyBac* transposon-mediated insertional mutagenesis method is useful for obtaining knockout mutants^16^, but few forward genetic tools are available for the generation of mutations, such as a mutation conferring drug resistance, via base substitution(s).

In general, the spontaneous mutation rates of eukaryotes are very low due to high nucleotide selectivity and the proofreading functions of DNA polymerase δ (Pol δ) and ε (Pol ε) during nuclear DNA replication as well as DNA repair. However, defects in any of these functions can yield mutator phenotypes with high mutation rates^17^,^18^. Mutator phenotypes can rapidly accumulate spontaneous mutations as the sources of both adaptive variation and a deleterious mutational load, which are involved in the emergence of drug resistance^19^ and tumourigenesis^18^, respectively. Thus, in a previous study, we developed a new genetic tool to facilitate the efficient generation of new mutants/traits and obtained a mutator malaria parasite (hereafter referred to as PbMut) using a rodent malaria parasite, *P. berghei*, by replacing the intrinsic Pol δ gene with a mutated gene that exhibits a defective 3′ → 5′ proofreading exonuclease activity^20^.

The specific type of mutator has different effects on tumourigenesis in humans and on the emergence of drug resistance in pathogenic bacteria because each mutator allele has a unique mutation spectrum^21^,^22^. Thus, we detected an ca. 86- to 90-fold increase in the base substitution rate in PbMut, but its mutation spectrum is unexplained. Therefore, elucidating the mutational trends in PbMut may help us to understand how the parasite acquires genetic variation to facilitate its adaptation to in-host environmental conditions, such as the presence of drugs, thereby allowing the construction and optimisation of the mutant generation system with PbMut. Most previous studies of mutators have been restricted to human cells and major model organisms. The findings obtained from *Plasmodium* species, which are phylogenetically distinct from the previously studied organisms, are also expected to facilitate our understanding of the general characteristics of Pol δ.

To obtain detailed insights into the mutation trends in the mutator malaria parasite, we experimentally evolved PbMut for over 24 weeks via mouse passage, and we identified the genome-wide accumulated mutations by NGS analysis. We demonstrated the existence of mutation bias in PbMut, as found in other Pol δ-mutator organisms. The base substitution rate was influenced by the sequence context, which affected the pattern of amino acid changes. Furthermore, the fitness of the Pol δ-mutator parasite was lower than that of the wild-type parasite, and the emergence of an antimutator allele was observed during the long-term passage of PbMut.

## Results

### Serial passage of parasites and the following NGS analysis

**Figure 1:**
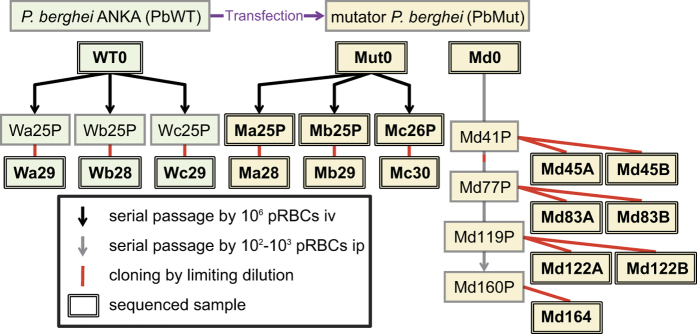
Schematic diagram showing the parallel passage lines of parasites. Three lines of PbWT parasites and four lines of PbMut parasites were maintained by serial passage in mice. Excluding the Md line, 10^6^ parasitised red blood cells (pRBCs) were injected intravenously (iv) into mice every 3 or 4 days in order to maintain the parasites. The Md line was maintained by weekly passage via intraperitoneal (ip) injection of 100 or 1,000 pRBCs into mice. The samples in double-lined squares were subjected to genome sequencing analysis.

To analyse the mutation trends in the mutator malaria parasite, the long-term accumulation of mutations followed by NGS analysis were performed with PbMut and its parental strain, *P. berghei* ANKA (clone 2.34) (hereafter referred to as PbWT). In a previous study, we produced one mutation accumulation line of PbMut (Md line in Fig. 1), and we reported mutations in two PbMut clones at the 122nd mouse passage^20^. In the present study, we produced three additional replicate passage lines (Ma, Mb and Mc) for PbMut and three (Wa, Wb, and Wc) for PbWT (Fig. 1). Genomic DNA was extracted from 10 PbMut and three PbWT clones after serial passages for over 24 weeks. The samples were designated with names, such as “Md45A,” where the initial two letters correspond to the passage line, the digits are the number of weeks the PbWT/PbMut parasites were maintained in mice, and “A” or “B” distinguishes clones passaged for the same period of weeks. To determine the diversity of the evolved PbMut populations, DNA was also extracted from population samples belonging to the Ma, Mb and Mc lines (Ma25P, Mb25P, and Mc25P in Fig. 1). The extracted DNA was analysed using an Illumina sequencing platform.

### Overview of mutations and base substitution rate

**Figure 2:**
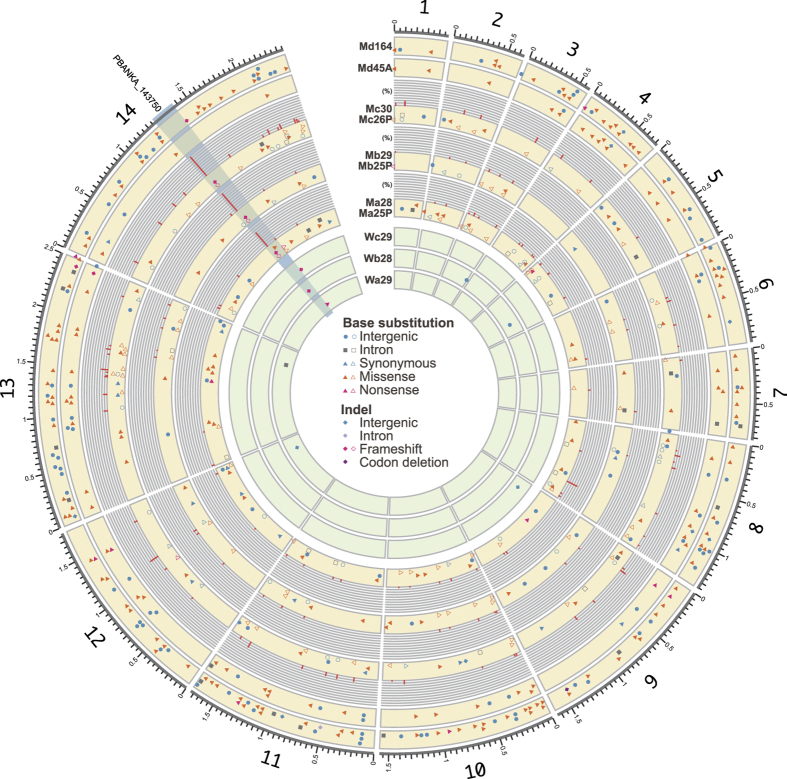
Distribution of mutations detected in PbWT and PbMut parasites. The outermost line shows the 14 chromosomes of *P. berghei*. The distributions of mutations detected in each sample are represented as follows: base substitutions in intergenic regions (light blue circles), base substitutions in introns (gray squares), synonymous mutations (light blue triangles), missense mutations (orange triangles), nonsense mutations (magenta triangles), indels in intergenic regions (light blue rhombuses), an indel in introns (a light purple rhombus), frameshift mutations (magenta rhombuses), and a codon deletion (a dark purple rhombus). Ma25P and Ma28, Mb29 and Mb25P, and Mc30 and Mc26P are grouped and displayed, where mutations from the population sample are represented by hollow glyphs and those from the clone sample are represented by solid glyphs. Bar charts outside the tracks show the distribution of mutations to indicate the frequencies of mutations detected from population samples. The AP2-G gene (PBANKA_143750) region is highlighted in light blue.

In the clones between weeks 28 and 30, we detected only 2–3 base substitutions in PbWT, whereas we detected 30–55 base substitutions in PbMut (Fig. 2, Supplementary Tables S1 and S2). However, the numbers of insertions and deletions (indels) were comparable in the PbMut (1–3 indels) and PbWT (1–2 indels) clones between weeks 28 and 30 (Supplementary Tables S1 and S3). We detected 84–223 base substitutions and 1–9 indels in the Md clones, where the number of mutations increased with the duration of the passage period. These observations suggest that the proofreading activity of *P. berghei* Pol δ contributes mainly to the reduced base substitution rate, which is consistent with previous studies of other organisms^21^,^23^. The base substitution rate (mean ± SE) in the nuclear DNA from PbMut (13.5 ± 1.7 × 10^−9^) was 36.5-fold higher than that from PbWT (0.37 ± 0.19 × 10^−9^) (P = 0.004; Welch’s *t*-test). This difference is lower than that (86- to 90-fold change) found in our previous study^20^, which was attributed to the fact that the base substitution rate of PbWT was higher than that of PbCtl with wild-type Pol δ (0.14 × 10^–9^ bp^−1^ day^−1^) in our previous study. In both studies, PbWT and PbCtl accumulated only several base substitutions, and thus more replicates and/or a longer passage period will be necessary to estimate the accurate base substitution rate of the parasite with wild-type Pol δ. The difference in base substitutions between PbMut and PbWT is considered to be several dozen times.

We detected 32–64 base substitutions in the PbMut population samples, and the frequencies of base substitutions in these populations ranged from 2% to 43% (Fig. 2, Supplementary Tables S1 and S2). Common substitutions were observed rarely in the PbMut clones and their derived populations. It is difficult to detect indels based on NGS data derived from populations. Therefore, we checked whether the indels detected in clones were confirmed based on the mapping data for population samples. The frameshift mutation at E352 in AP2-G (PBANKA_143750) was commonly observed in three PbMut population samples (Ma25P, Mb25P, and Mc26P) at a high frequency (Fig. 2 and Supplementary Table S3).

### Mutation spectrum of base substitutions

**Figure 3:**
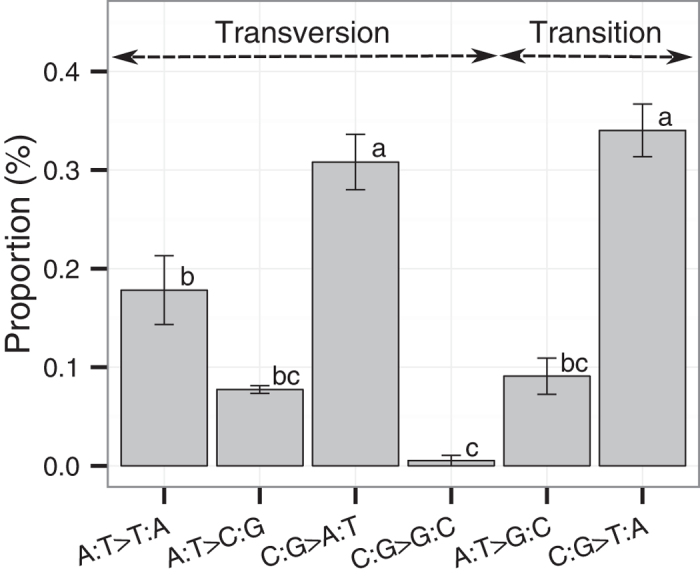
Relative mutation rates of six possible base substitutions in PbMut. This chart was generated with the data obtained from Ma28, Mb29, Mc30, and Md45A. There was a statistically significant difference between base substitution types with different letters (Holm-adjusted P < 0.05; Student’s *t*-test). Error bars represent standard errors.

The genome of *P. berghei* has a high AT content (% AT = 77.3, estimated from the reference genome). In order to determine the mutation spectrum under equal AT and CG contents, the relative rates of the six possible base substitution types were calculated after normalising the number of base substitutions at AT sites, as described previously^24^. We found that the mutation spectrum of PbMut was not uniform (Fig. 3). The proportions of C:G to A:T and C:G to T:A substitutions were significantly higher than the other substitutions (Holm-adjusted P < 0.05; Student’s *t*-test), and these two patterns accounted for 64.8% of the total. In addition, C:G to G:C transversions were rare, thereby indicating that the base substitutions at CG sites were strongly biased towards AT. At AT sites, the proportion of A:T to T:A transversions appeared to be higher than that of A:T to C:G and A:T to G:C substitutions, but these differences were not significant.

The mutation spectrum of PbMut was highly biased towards C/G to A/T, and thus the AT content was predicted to have increased from the initial value of 77.3%. The expected equilibrium AT content (AT_eq_) was estimated based on the mutation spectrum^25^. The calculated mean AT_eq_ was 79.9%, which approximated to the current AT content of the *P. falciparum* genome (% AT = 80.6)^6^.

### Sequence context-dependent mutation rate

**Figure 4:**
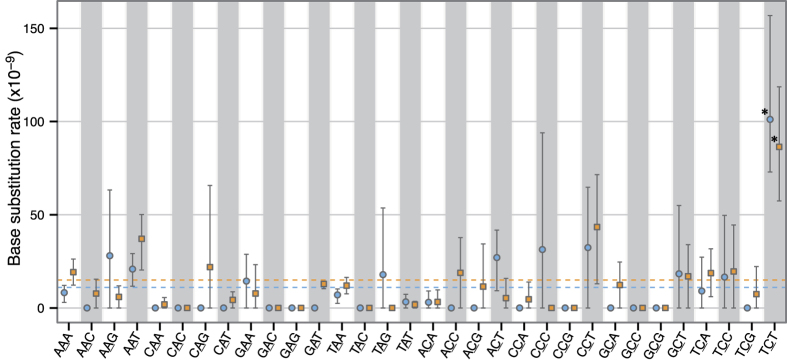
Sequence context-dependent mutation rate in PbMut. The sequence context-dependent mutation rates for intergenic (light blue circles) and genic (orange squares) regions were calculated with the data obtained from Ma28, Mb29, Mc30, and Md45A. The orange and light blue dashed lines indicate the overall mutation rates in intergenic (11.2 ± 0.7 × 10^−9^ base^−1^ day^−1^) and genic (15.0 ± 2.3 × 10^−9^ base^−1^ day^−1^) regions, respectively. A statistically significant elevated mutation rate was observed at TCT compared with the average mutation rate in each region (*p < 0.05; Welch’s *t*-test). Error bars represent the bootstrap-estimated 95% confidence intervals.

To determine whether the base substitution rate was influenced by the sequence context, we analysed the context-dependent mutation rate based on the trinucleotide sequence, including the substituted sites and their flanking 3′ and 5′ bases. We ignored the strand orientation and integrated two complementary sequences into one sequence category, where the central base was A or C (e.g., CAT and ATG were categorised as CAT), thereby yielding 32 sequence categories. The context-dependent mutation rate was calculated for intergenic and genic (exon and intron) regions (Fig. 4). There were significant differences in the context-dependent mutation rates in both regions (intergenic regions, P = 5.2 × 10^−5^; genic regions, P = 3.1 × 10^−4^; Kruskal–Wallis test). In particular, the base substitution rates at TCT were 9.0- and 5.8-fold higher for intergenic and genic regions, respectively, compared with the corresponding overall mutation rates (intergenic regions, P = 0.048; genic regions, P = 0.031; Welch’s *t*-test). There was no significant difference between the mutation rates of C:G to A:T and C:G to T:A substitutions at TCT (P = 0.6508; Welch’s *t*-test) (Supplementary Fig. S1). Our sample size, however, was limited and a more detailed study with more passage replicates will be necessary to elucidate thoroughly the mutation spectrum of PbMut.

The distribution of context-dependent substitution rates probably affects the trends in the changes of amino acids. We detected 16 nonsense mutations in PbMut, among which 68.8% (11/16) occurred at Glu codons (Table 1). We scanned the codon distribution in *P. berghei* nuclear DNA and found that 17.2% of the possible codons causing a nonsense mutation with a single base substitution were Glu codons (Supplementary Fig. S2). Nonsense mutations in PbMut occurred significantly more frequently at Glu codons compared with their frequency in nuclear DNA (P = 1.4 × 10^−6^; Fisher’s exact test) (Supplementary Fig. S2). In a previous study, a similar observation was made for proofreading-deficient human Pol ε, where the mutation rate was markedly elevated at TCT^26^. Further, it was also shown that Glu codons (GAG and GAA) can form the hotspot motif TCT (AGA) if they are preceded by adenine (AGAG and AGAA), whereas a G to T mutation at the first base in Glu codons generates stop codons (UAG and UAA). The elevated mutation rate at TCT in PbMut is equally likely to contribute to increased nonsense mutations at Glu codons.

### Accumulated mutations in parallel passage lines

**Figure 5:**
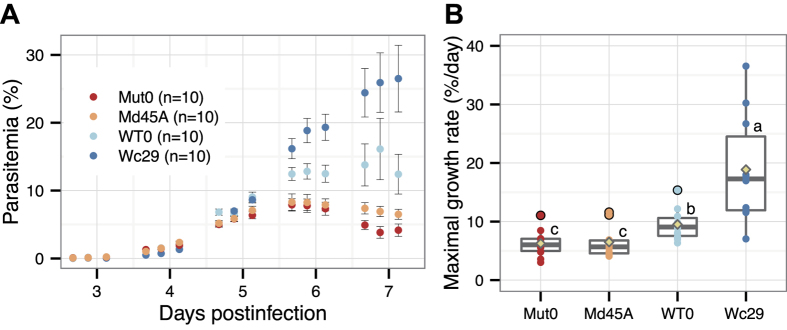
Growth of ancestral and evolved parasites. (**A**) Parasite growth was monitored for two mutator clones (Mut0 and Md45A) and two wild-type clones (WT0 and Wc29). Parasitemia was derived based on the mean of 10 mice (±SE). (**B**) Maximal growth rates (% day^−1^) were estimated between days 3 and 6. Individual circle dots represent the maximal growth rate of parasites observed in individual mice, and yellow rhombus dots represent the mean. Boxplots show the distribution of data, where the middle, top, and bottom lines on a box represent the median and 75th and 25th percentiles of the data, respectively. There was a statistically significant difference between clones with different letters (Holm-adjusted P < 0.05; Wilcoxon rank-sum test).

Previous studies have demonstrated that parallel evolution can occur among replicate lineages of microbes^27^,^28^. We detected 289 genes with amino acid changes, among which 14 were shared in parallel passage lines (Table 2). In addition, 9/14 genes were proteins with unknown functions. It should be noted that truncation of the AP2-G gene (PBANKA_143750) due to nonsense or frameshift mutations was detected repeatedly in all of the samples that we analysed. AP2-G is one of the transcription factor AP2 family proteins in *Plasmodium* species, and it is considered to be a master regulator involved in the development of gametocytogenesis^29^,^30^. The emergence of gametocyte non-producers has been reported during the asexual multiplication of *P. berghei* in mice^31^. In a previous study, we also observed a frameshift mutation at P2298 in AP2-G in two PbMut clones during the 122nd mouse passage (Md122A and Md122B) (Supplementary Fig. S3D), in which we could not detect exflagellation or ookinete formation^20^. In the present study, we detected a shared frameshift mutation at E352 in the AP2-G gene among Ma28, Mb29, and Mc30 (Supplementary Table S3). Sanger sequencing analyses demonstrated that the E352 mutation had already emerged at around the sixth week during each passage (Supplementary Fig. S3A–C), and NGS analyses of Ma25P, Mb25P, and Mc26P samples showed that this mutation was present in 70–95% of each population at the 25th–26th mouse passage (Fig. 2). We could not detect the frameshift mutation based on the NGS data for the founder mutator clone (Mut0), which suggests that the frameshift mutation was already present before serial passages but below the detection limit. It was probably present in less than 1.4% of the population because the number of mapped reads at E352 in Mut0 was 72. It is assumed that the frameshift mutation emerged during multiplication in the series of cloning procedures. We also detected another truncation of AP2-G due to a nonsense mutation at E2264 in Ma25P, which was present in 3.5% of the population (Fig. 2 and Supplementary Table S2). Moreover, unique knockout mutations in AP2-G were observed in PbWT clones (G2044 nonsense, K290 frameshift, and K2188 frameshift mutations in Wa29, Wb28, and Wc29, respectively), thereby indicating that these mutations emerged individually in each passage line (Table 2 and Supplementary Tables S2 and S3). Sanger sequencing of Wa25P, Wb25P, and Wc25P showed that these variants accounted for large proportions of the populations (Supplementary Fig. S3E–G). To determine the influence of the accumulated mutations on asexual multiplication, we estimated the maximal growth rates during the course of infection with Mut0, Md45A, WT0, and Wc29 (Fig. 5A–B). Md45A contained 87 mutations, but there were no mutations in the AP2-G gene. By contrast, Wc29 had only two indels (one in an intergenic region and the other in the AP2-G gene with a frameshift mutation) (Supplementary Table S2). We used the gcFitModel function in the grofit R package^32^ to conduct growth curve fitting and to estimate the maximal growth rate (Fig. 5B). In PbWT, the maximal growth rate (mean ± SE) of Wc29 (18.9 ± 3.0% day^−1^) was about two times higher than that of WT0 (9.5 ± 0.9% day^−1^) (Holm-adjusted P = 0.016; Wilcoxon rank sum test), whereas in PbMut, there was no significant difference between those in Mut0 (6.3 ± 0.7% day^−1^) and Md45A (6.5 ± 0.9% day^−1^) (Holm-adjusted P = 0.97; Wilcoxon rank sum test). An enhanced growth rate was observed in Wc29, thereby indicating that the knockout of AP2-G yielded increased fitness in the asexual stage. However, there appeared to be no increase in the fitness of Md45A, which accumulated 54 missense, two nonsense, and one frameshift mutations. It should also be noted that the maximal growth rate of WT0 was significantly higher than that of Mut0 (Holm-adjusted P = 0.021; Wilcoxon rank sum test), which suggests that the defective proofreading by Pol δ led to the decline in fitness.

### Emergence of an antimutator mutation

We detected the E794K mutation in Pol δ from Ma25P, which accounted for 5% of the population (Supplementary Table S2). Interestingly, this mutation was equivalent to the E800K mutation in yeast Pol δ identified in a study of mutator yeast (Supplementary Fig. S4) 33. E800 in yeast Pol δ is adjacent to E802, which is the third metal-binding site related to modulation of the catalytic efficiency^34^, and the E800K mutation reduced the capacity of mutator yeast to mutate and escape from replication-error-induced extinction^33^,^35^. Therefore, the E794K mutation in *P. berghei* Pol δ appears to be an antimutator substitution.

### Detrended correspondence analysis of RNA-Seq data

**Figure 6:**
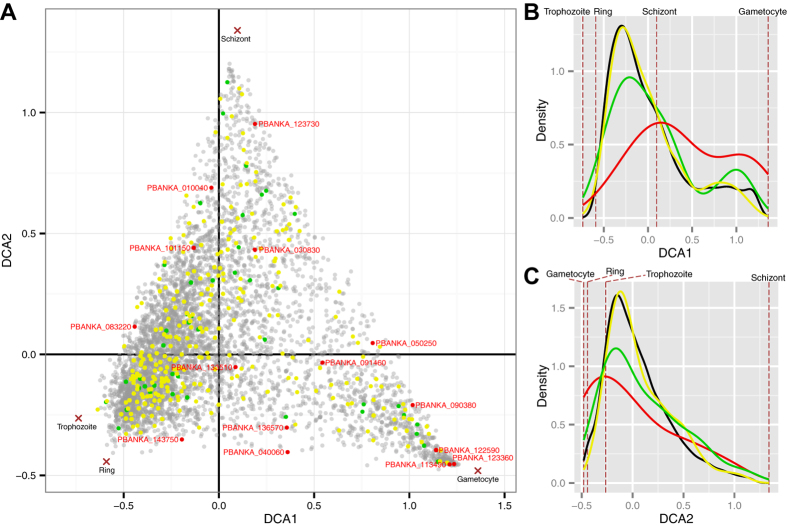
Detrended correspondence analysis based on RNA-Seq data for *P. berghei* genes in four developmental stages. (**A**) Circle dots are *P. berghei* genes, and cross marks denote four developmental stages (ring, trophozoite, schizont, and gametocyte). Mutated genes in PbMut are highlighted as follows: synonymous-mutated genes (green), missense-mutated genes (yellow), and nonsense-mutated genes (red). The names of nonsense-mutated genes are also shown in red letters. (**B**,**C**) Kernel density plots showing the distributions of dots along the DCA1 and DCA2 axes. Green, yellow, red, and black lines denote the densities of synonymous-mutated, missense-mutated, nonsense-mutated, and all genes, respectively.

In total, we detected 584 base substitutions based on the PbMut samples. To visualise the relationships between the mutated genes and their expression patterns during *P. berghei* development, we downloaded RNA-Seq data (1037cl1cl15cy1 profiles) for the four developmental stages (ring, trophozoite, schizont, and gametocyte) from PlasmoDB (http://plasmodb.org/plasmo/)^10^, which we analysed by detrended correspondence analysis (DCA) (Fig. 6). The distribution of nonsense-mutated genes along DCA1 differed significantly from that of all genes (P = 0.01422; Kolmogorov–Smirnov test). Nonsense mutations tended to occur mainly in genes expressed during the gametocyte stage, whereas they tended to occur infrequently in genes expressed mainly during the ring and trophozoite stages, thereby suggesting that the nonsense mutations occurred in genes under relaxed constraints during asexual multiplication. However, the distributions of missense- and synonymous-mutated genes did not differ significantly from those of all genes along DCA1 (missense vs. overall, P = 0.6536; synonymous vs. overall, P = 0.2941; Kolmogorov–Smirnov test), thereby suggesting that these mutation types occurred randomly regardless of the gene expression trends. There was no significant difference between the distribution of all genes and that of synonymous-, missense-, or nonsense-mutated genes along DCA2.

## Discussion

In this study, we determined the biased mutation spectrum of PbMut, in which the predominant mutation comprised changes from C/G to A/T (Fig. 3). The existing data available across prokaryotes and eukaryotes show that the prevailing mutation bias is generally in the direction of AT^24^,^25^, but it is remarkable that a biased mutation trend towards AT was observed in PbMut with a highly AT-biased genome (% AT = 77.3). *P. falciparum* has an extremely AT-rich genome (% AT = 80.6), and an overall mutation trend towards increasing AT richness was demonstrated based on the sequence analysis of 10 genetic loci^36^. By contrast, the genome sequencing of 25 culture-adapted *P. falciparum* isolates from Senegal showed that the nucleotide composition was at or near equilibrium^37^. The AT content of the *P. berghei* genome is high but lower compared with that of the *P. falciparum* genome; therefore, the *P. berghei* genome appears to have sufficient capacity to accommodate C/G to A/T changes. Interestingly, the expected equilibrium AT content calculated from the mutation spectrum of PbMut was 79.9%, which is approximately the current AT content of *P. falciparum*, thereby implying that an AT content of about 80% might be the limit for *Plasmodium* genomes.

Changes from C:G to A:T and C:G to T:A were observed at similar frequencies in the PbMut mutation spectrum (Fig. 3). DNA lesions caused by oxidation and the deamination of bases are the major factors that induce DNA replication errors. 7,8-dihydro-8-oxoguanine is well known to be the product of oxidation damage to guanine, and it can mismatch with adenine, thereby leading to C:G to A:T transversions^38^. The deamination of 5-methyl-cytosine (me^5^C) produces thymine, which can lead to C:G to T:A transitions^39^. The extent of DNA damage in the *P. berghei* genome is unknown, but the high frequency of C/G to A/T changes suggests that replication errors caused by DNA damage may play a major role in the formation and maintenance of the AT-rich *P. berghei* genome. Ponts *et al*. reported that *Plasmodium* parasites possess single functional DNA methyltransferases that mediate cytosine methylation, and 0.67% of the total cytosines are methylated in the *P. falciparum* genome^40^. They also demonstrated that the positions surrounding conserved me^5^C residues exhibit a significant preference for thymine. Interestingly, PbMut exhibited a markedly increased mutation rate at TCT (Fig. 4). The motif in which base substitutions occur frequently is unknown, e.g., TCT and/or AGA, but these observations indicate that the methylation of cytosines surrounded by thymines might be involved in the formation of the hotspot motif TCT. Thus, a high mutation rate in terms of C:G to T:A transitions was expected to occur at TCT, but there was no significant difference in the frequencies of C:G to T:A and C:G to A:T changes (Supplementary Fig. S1). However, our sample size was small and a more detailed analysis with more replicates will be necessary to conclusively elucidate the mutation spectrum at TCT. Interestingly, a markedly elevated mutation rate at TCT has also been reported in cancer genomes with proofreading-deficient Pol ε^26^,^41^,^42^. The proofreading-deficient human Pol ε exhibits a preference for TCT to TAT transversions *in vitro*^26^, and thus the deamination of me^5^C does not appear to be involved in the elevated mutation rate in tumours. Therefore, the high mutation rate at TCT in PbMut may be attributable to multiple factors, which should be clarified in future research.

The number of accumulated mutations during long-term passages differed greatly between PbWT and PbMut (Fig. 2). When we compared the growth rates of the ancestral clones with those of the evolved clones to evaluate the effects of the accumulated mutations on fitness, we found that the growth rate of Wc29 was significantly higher than that of WT0, whereas there was no difference between Mut0 and Md45A (Fig. 5). Md45A had 87 mutations, but most were weakly deleterious or neutral, and they had little impact on fitness. Wc29 had only two indels, one of which was in an intergenic region and was a neutral mutation. Therefore, the other indel, which was a knockout mutation in the AP2-G gene, must have boosted asexual multiplication of Wc29. These observations suggest that a single, highly effective mutation influences parasite growth rather than many low-impact mutations. An increased growth rate during the asexual stage was also reported in a previous study in which the AP2-G gene of *P. berghei* was artificially disrupted^30^. Nonsense or frameshift mutations in the AP2-G gene occurred in all of the passage lines, thereby indicating that the knockout mutation in the AP2-G gene confers strong growth advantages in the parasites during the blood stage, but at the cost of losing the ability to form gametocytes. The knockout mutation of AP2-G appears to be fixed immediately within the *P. berghei* population during long-term *in vivo* mouse passage in the laboratory, unless it is excluded stochastically from the population when it first occurs. The Md passage line required longer before the first detection of the AP2-G knockout mutation than did the other lines, which was around the 112th mouse passage (Supplementary Fig. S3D). There was a severe bottleneck during each mouse passage in the Md line; therefore, even if AP2-G knockout mutations occurred spontaneously early during the course of passage, they appeared to have been excluded stochastically from the population.

In the present study, we unexpectedly found that the growth rates of PbMut clones were significantly lower than those of PbWT clones (Fig. 5B). However, we found no differences in the growth rates among PbWT, PbMut, and PbCtl, which was the control *P. berghei* transfected by wild-type Pol δ and human dihydrofolate reductase (hDHFR)^20^. However, we consider that the observations in our previous study are consistent with those in the present study because the PbMut clones (Md122A and Md122B) used in the growth assay in the previous study had a frameshift mutation in AP2-G (Supplementary Table S3), and thus, their growth rates should have been accelerated, as discussed above. Three reasons may explain the decreased growth rate of PbMut: (1) mutated Pol δ has a direct fitness cost; (2) the increased mutation rate generates more deleterious mutations, thereby decreasing fitness indirectly; and (3) the hDHFR gene (*hdhfr*) introduced into the *P. berghei* genome with the mutated Pol δ as a drug selectable marker has a fitness cost. A previous study showed that a recombinant *P. berghei* expressing green fluorescent protein and *hdhfr* genes had similar growth characteristics to the wild-type parasite^43^, which suggests that the fitness cost of *hdhfr* is negligible. Therefore, we suggest that the decreased growth rate of PbMut was not attributable to *hdhfr* but instead was a result of the direct or indirect effects of the mutated Pol δ. Pol δ is involved in DNA repair as well as DNA replication^44^, so eliminating the proofreading activity of Pol δ might influence the stability of the genome.

Interestingly, the appearance of the putative antimutator mutation E794K in Pol δ was identified during long-term passages of the Ma line. To the best of our knowledge, this is the first observation of the antimutator mutation in malaria parasites. The analogous mutation (E800K) in yeast Pol δ caused a 33-fold decrease in the rate of mutation in mutator yeast^35^. If the strength of the antimutagenic effect of E794K in *P. berghei* Pol δ is comparable with that of E800K in yeast Pol δ, the mutation rate of PbMut-E794K may be similar to that of PbWT. These observations of a common antimutator mutation in phylogenetically distinct organisms, such as yeast and a malaria parasite, suggest that the corresponding mutation in eukaryotic Pol δ at E800K in yeast Pol δ may have a universal antimutagenic effect.

Our results, in terms of the decreased growth rate of PbMut and the emergence of the antimutator mutation, have implications for the behavior of Pol δ-mutator parasites in the field. Pol δ-mutator malaria parasites in the field have not been reported previously. Even if the Pol δ-mutator parasite did emerge in the field, the wild-type parasite would outcompete the mutator parasite, making it impossible for the mutator parasite to establish itself at the population level. It has been reported that the mutator is involved in the acquisition of drug resistance in bacteria^45^,^46^, but this might not be the case with Pol δ-mutator malaria parasites. A recent study showed that the mutation rates of *P. falciparum* isolates from Southeast Asia, where drug resistance has emerged repeatedly, were not significantly higher compared with those of isolates from West Africa^47^. However, our knowledge of mutator malaria parasites is still inadequate. Thus, it should be useful to investigate the fitness costs of various mutator phenotypes and the competition between mutator and wild-type parasites under drug pressure.

Finally, we discuss the properties of PbMut in terms of its possible applications as a genetic tool. NGS analyses of PbMut samples after serial passages demonstrated that there were no fixed mutations within the populations except for knockout mutations in AP2-G (Fig. 2), and few mutations were shared between the clone and the population samples (Supplementary Table S2). These results reflect the great number of variants accumulated within populations after long-term passages, most of which were present below detectable levels. In addition, DCA plots using RNA-Seq data showed that missense mutations occurred randomly in genes regardless of their expression trends (Fig. 6), thereby suggesting that we had constructed a parasite library containing various mutants with dozens of missense mutations. We found that gametocyte non-producers were frequently fixed within populations after long-term mouse passages. However, a mouse passage combined with a mosquito passage would be effective for suppressing the loss of the capacity to form gametocytes in future studies. Alternatively, the gametocyte non-producer phenotype appeared to the result of AP2-G gene knockout alone, so the ability to form gametocytes can be readily recovered by CRISPR/Cas editing of the AP2-G gene^48^. More importantly, the mutation bias and the biased sequence-context mutation rates are likely to affect the variety of mutants generated from PbMut. In fact, a high occurrence of nonsense mutations at Glu codons was observed in PbMut, which was attributed to the elevated mutation rate at TCT (Supplementary Fig. S2). It has been reported that different mutator *E. coli* strains can generate distinct fitness distributions for antibiotic-resistant mutants according to their mutation spectra^22^. Therefore, PbMut and other types of mutators distinct from PbMut generated from alternative approaches may be useful for developing a system to generate various mutated phenotypes/traits. The base substitution bias of PbMut may be disadvantageous in terms of generating more diverse mutants, but we consider that this feature may be advantageous from different perspectives. For example, Shinbrot *et al*.^26^ used observations of strand-specific mutation patterns that emerged from Pol ε mutants to demonstrate the leading-strand-specific replication of human Pol ε^26^. If the hotspot motif of PbMut is completely identified (i.e., TCT or AGA), detailed analyses of mutational hotspots in PbMut will enable us to map the lagging/leading synthesis regions in the *P. berghei* genome, ultimately leading to the elucidation of the distribution of replication origins that remain unknown in the genomes of *Plasmodium* parasites.

## Materials and Methods

### Parasites and mutation accumulation lines

The PbMut used in this study was previously generated from *P. berghei* ANKA clone 2.34^20^. Briefly, two amino acids critical for the 3′ → 5′ proofreading exonuclease activity in Pol δ were replaced with alanine. We obtained the founder clones WT0, Mut0, and Md0 by recloning with a limiting dilution of *P. berghei* ANKA clone 2.34 and PbMut prior to conducting the serial mouse passages. We created four (Ma, Mb, Mc, and Md) and three (Wa, Wb, and Wc) mutation accumulation lines for PbMut and PbWT, respectively (Fig. 1). Excluding the Md line, we intravenously injected 10^6^ parasitised red blood cells (pRBCs) into female ddY mice (6–10 weeks old; Japan SLC) every 3 or 4 days to maintain the parasites. The Md line was maintained by weekly passage via intraperitoneal injection of 100 or 1,000 pRBCs into mice^20^. During serial passage, the cloning of parasites by limiting dilution was achieved in the 53rd week of passage for the Md line. All animal research and experimental protocol were approved and conducted in accordance with the guidelines and regulations of the Animal Ethical Committee of the Research Institute for Microbial Diseases, Osaka University, and the Animal Care Committee of Kyorin University School of Medicine. The assigned license numbers are no. 151 (Kyorin University) and no. DOUBI-H20-070-0 (Osaka University), respectively.

### DNA extraction and high-throughput genome sequencing

Ten clones from the PbMut parasites and three clones from the PbWT parasites were obtained by limiting dilution (Fig. 1). We intravenously injected 10^6^ infected erythrocytes from each clone and each PbMut population into 20 female ddY mice. On day 4 post infection, blood was collected by cardiac puncture to extract parasite DNA. Infected blood was diluted three times with HEPES buffered saline (HBS: 140 mM NaCl, 5 mM KCl, and 10 mM HEPES-NaOH at pH 7.2) and filtered twice through Plasmodipur filters (EuroProxima) to deplete the host leukocytes. The filtrate was centrifuged for 5 min at 3,000 rpm, and the supernatant was discarded. The pellet was suspended at 4 °C in 0.5% saponin in HBS to lyse the erythrocytes. The lysate was then centrifuged for 15 min at 8,000 rpm and washed four times with HBS. The pellet was subjected to DNA extraction with a QIAamp^®^ DNA Blood Mini Kit (Qiagen), according to the manufacturer’s instructions. The extracted genomic DNA was sequenced using an Illumina HiSeq 2000 or an Illumina Genome Analyzer II, from which we obtained 1.1–1.5 Gb and 4.2–5.0 Gb sequence data from the clone and population samples, respectively (Supplementary Table S1). These sequence data corresponded to >60- and >230-fold redundancy for the clone and population samples, respectively, relative to the reference genome sequence of *P. berghei* ANKA (18.2 Mb). Low quality reads and reads derived from host DNA were excluded, as described previously^20^.

### Read mapping and mutation identification

The paired-end reads were mapped to the reference genome sequence downloaded from the Wellcome Trust Sanger Institute (https://www.sanger.ac.uk/) using BWA version 0.7.5a^49^, and duplicate sequences were removed using Picard tools version 1.96 (http://sourceforge.net/projects/picard/). Local realignment around indel sites and base quality recalibration were conducted using the Genome Analysis Toolkit (GATK) version 2.7.4^50^. Subsequently, variant calling based on the alignments of clones was performed using two pipelines, i.e., one with VarScan2 version 2.3.6^51^ and the other with GATK. The alignments were converted into mpileup files using SAMtools^52^. Base substitutions and indels were called from mpileup files with VarScan2 using the following conditions: ≥10 base coverage; mutation frequency criteria of ≥80% and ≥50% for base substitutions and indels, respectively; and a minimum PHRED quality score of 20. Base substitutions and indels were also called by GATK UnifiedGenotyper using a minimum PHRED quality score of 20. Detected variants were confirmed by visual inspection of mapping data with the Integrated Genome Viewer^53^. We validated all indels, all base substitutions causing nonsense mutations, and all base substitutions detected using either of the two pipelines by Sanger sequencing (Supplementary Tables S2–S4). We also sequenced the ancestral clones (WT0, Mut0, and Md0) of each passage line to identify only the mutations accumulated during serial passages, and their variants were excluded from the subsequent analysis. The detected variants were annotated using SnpEff version 3.6c^54^. To summarise the distribution of mutations, a Circos plot was generated using the Circos program^55^.

The detection of base substitutions based on the alignments of populations was performed using LoFreq version 2.0.0 with the default settings^56^. Visual inspection and annotation of detected base substitutions were performed as described above.

### Calculation of the base substitution rate, the relative rates of six possible base substitutions, and the expected equilibrium AT content

The base substitution rate (μ_bs_) was calculated according to the equation: μ_bs_ = m/(nT), where m is the number of mutations detected, n is the number of nucleotide sites mapped by ≥10 reads, and T corresponds to the number of passage days^57^. The average base substitution rate was calculated using the data for clones isolated from parallel passage lines; i.e., only data from Ma28, Mb29, Mc30 and Md45A were used for calculating the rate for PbMut. In the same manner, the average sequence context-dependent mutation rate for PbMut was calculated.

We calculated the relative rates of six possible base substitutions according to Hershberg and Petrov (2010)^24^. Briefly, the numbers of mutations that occurred at A/T sites were normalised by multiplying them by #CG_sites_/#AT_sites_, where #CG_sites_ and #AT_sites_ are the total numbers of C/G or A/T sites in the current *P. berghei* nuclear genome, respectively. In order to calculate the relative rates, the normalised counts of mutations at A/T sites and unaltered counts of mutations at C/G sites were divided by the sum of these counts.

The expected equilibrium AT content (AT_eq_) was calculated according to Lynch (2007)^25^ using the following equation: AT_eq_ = *r*_(CG→AT)_/(*r*_(AT→CG)_ + *r*_(CG→AT)_), where *r*_(AT→CG)_ is the mutation rate from A/T to C/G, whereas *r*_(CG→AT)_ is that from C/G to A/T. *r*_(CG→AT)_ was calculated using the following equation: *r*_(CG→AT)_ = *f*_(CG→AT)_/(1 – *p*). *r*_(AT→CG)_ was calculated using the following equation: *r*_(AT→CG)_ = *f*_(AT→CG)_/*p*, where *f*_(CG→AT)_ and *f*_(AT→CG)_ are the observed frequencies of C/G to A/T and A/T to C/G substitutions, respectively, and *p* is the current AT content (% AT).

### Growth assay for parasites

To assess the difference in growth between the founder and evolved clones, four groups of female BALB/c mice (6 weeks old; Clea Japan, Inc.) each with 10 mice per group were inoculated intravenously with 10^4^ erythrocytes infected with PbMut clone Mut0, PbMut clone Md45A, PbWT clone WT0, or PbWT clone Wc29. Parasitemia was monitored three times each day between days three and seven post-infection. The parasite density was estimated by microscopically counting the number of infected erythrocytes per 3,000 erythrocytes based on Giemsa-stained thin blood smears.

### Statistical analysis

All of the statistical tests in this study were conducted in R^58^. P < 0.05 was considered statistically significant. Graphs were generated using the ggplot2 R package^59^. DCA was performed using the vegan R package^60^ based on the expression data for 4896 protein-coding genes, which we obtained after excluding genes with low expression levels (reads per kilobase of transcript per million mapped reads, RPKM < 1) at all stages.

### Data access

The raw sequence reads used in this study are available from the DDBJ Sequence Read Archive (DRA) under accession numbers DRA000656 and DRA004715.

## Additional Information

**How to cite this article**: Honma, H. *et al*. Mutation tendency of mutator *Plasmodium berghei* with proofreading-deficient DNA polymerase δ. *Sci. Rep.* **6**, 36971; doi: 10.1038/srep36971 (2016).

**Publisher’s note**: Springer Nature remains neutral with regard to jurisdictional claims in published maps and institutional affiliations.

## Supplementary Material

Supplementary Information

Supplementary Information

## Figures and Tables

**Table 1:** Nonsense mutations in *P. berghei* wild-type and mutator clones.

Gene name	Product (total amino acid length)	AA position	Trinucleotide^a^	Sample
PBANKA_010040	reticulocyte binding protein, putative (613)	E293	AGA	Mb25P
PBANKA_030830	GAF domain-related protein, putative (1175)	K726	AAA	Md45B
PBANKA_040060	BIR protein(PIR protein) (312)	E47	TCA	Md164
PBANKA_050250	serine/threonine protein phosphatase, putative (2095)	G372	CCT	Ma28
PBANKA_083220	conserved *Plasmodium* protein, unknown function (300)	E87	AGA	Md122B
PBANKA_090380	serine/threonine protein kinase, putative (965)	E677	TGA	Md45A, Md45B, Md83A, Md83B, Md122A, Md122B, Md164
PBANKA_091460	RNA (uracil-5-)methyltransferase, putative (790)	K230	TTT	Ma28
PBANKA_101150	conserved *Plasmodium* protein, unknown function (4789)	E4717	AGA	Md83B
PBANKA_102460	liver-specific protein 1 (3254)	E3077	AGA	Md164
PBANKA_113490	zinc finger protein, putative (870)	E336	AGA	Md83A, Md83B, Md122A, Md122B, Md164
PBANKA_122590	conserved *Plasmodium* protein, unknown function (2057)	E245	AGA	Mc26P
PBANKA_123360	secreted ookinete protein, putative (205)	E144	TCT	Md122A
PBANKA_123730	inner membrane complex protein, putative (486)	S465	TCA	Md45A, Md45B, Md83A, Md83B, Md122A, Md122B, Md164
PBANKA_133510	conserved *Plasmodium* protein, unknown function (1899)	E1071	TCT	Ma28
PBANKA_136570	BIR protein (PIR protein) (322)	S220	TCA	Md164
PBANKA_143750	transcription factor with AP2 domain(s), putative (2339)	G2044	AGG	Wa29
PBANKA_143750	transcription factor with AP2 domain(s), putative (2339)	E2264	AGA	Ma25P

^a^The substituted base and its flanking bases are shown.

**Table 2:** Genes with missense, nonsense, and frameshift mutations in parallel passage lines.

Gene name	Product	Annotated GO function (GO ID)	Mutation (passage line)^a^
PBANKA_010040	reticulocyte binding protein, putative	null	E293* (Mb), E293K (Mc)
PBANKA_021160	conserved *Plasmodium* protein, unknown function	null	K68N (Mc), Q151K (Md), I315F (Md)
PBANKA_031160	conserved *Plasmodium* protein, unknown function	null	I698T (Mb, Mc)
PBANKA_050470	conserved *Plasmodium* protein, unknown function	null	L328I (Mc), I329F (Md)
PBANKA_083150	conserved *Plasmodium* protein, unknown function	null	A1711T (Md), L3595M (Mc)
PBANKA_091460	RNA (uracil-5-)methyltransferase, putative	RNA methyltransferase activity (GO:0008173)	E19K (Md), K230* (Ma)
PBANKA_100160	SET domain protein, putative	protein binding (GO:0005515)	Q326H (Mc), N507I (Mb)
PBANKA_102460	liver-specific protein 1	null	E978K (Mc), N1948T (Ma), E3077* (Md)
PBANKA_103740	conserved *Plasmodium* protein, unknown function	null	D189E (Mc), G202V (Mb)
PBANKA_122830	conserved *Plasmodium* protein, unknown function	electron carrier activity (GO:0009055), oxidoreductase activity (GO:0016491)	F434V (Ma), D2724Y (Mb)
PBANKA_131690	conserved *Plasmodium* protein, unknown function	null	F78I (Ma), I902F (Mc)
PBANKA_134340	conserved *Plasmodium* protein, unknown function	null	S1122Y (Mb), Q1687H (Md), N2067H (Md)
PBANKA_141960	conserved *Plasmodium* protein, unknown function	null	I1064T (Ma), K1072Q (Md), N1510Y (Md)
PBANKA_143750	transcription factor with AP2 domain(s) (AP2-G)	sequence-specific DNA binding transcription factor activity (GO:0003700)	K290fs (Wb), E352fs (Ma, Mb, Mc), N833Y (Ma), G2044* (Wa), K2188fs (Wc), Q2262K (Ma), E2264* (Ma), P2298fs (Md)

^a^,*Indicates a nonsense mutation, and fs indicates a frameshift mutation.
