# Supplementary material for: Mutation tendency of mutator Plasmodium berghei with proofreading-deficient DNA polymerase δ
Source: Sci Rep. 2016 Nov 15;6:36971. doi: 10.1038/srep36971 (PMC5109483; doi:10.1038/srep36971)
Supplement: Supplementary Information [file srep36971-s1.pdf]

**Mutation tendency of mutator *Plasmodium berghei* with proofreading-deficient DNA polymerase δ**

Hajime Honma[1], Mamoru Niikura[2], Fumie Kobayashi[2], Toshihiro Horii[3], Toshihiro Mita[4], Hiroyoshi Endo[1] & Makoto Hirai[4]

[1]Department of International Affairs and Tropical Medicine, Tokyo Women's Medical University, 8-1 Kawada-cho, Shinjuku, Tokyo, Japan, [2]Department of Infectious Diseases, Kyorin University School of Medicine, 20-2, Shinkawa 6, Mitaka-city, Tokyo, Japan, [3]Department of Molecular Protozoology, Research Institute for Microbial Diseases, Osaka University, 3-1 Suita, Osaka, Japan, [4]Department of Molecular and Cellular Parasitology, Juntendo University, 2-1-1 Hongo, Bunkyo, Tokyo, Japan

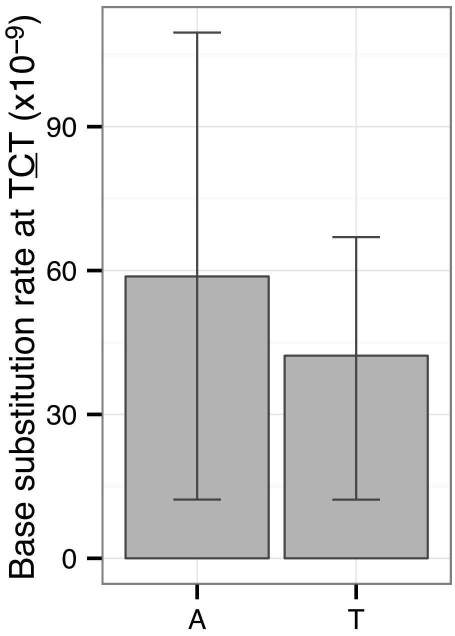


**Supplementary Figure S1. Base substitution patterns at the TCT motif in intergenic regions.** The mean mutation rates from cytosine to adenine or thymine on the horizontal axis were calculated with the data obtained from Ma28, Mb29, Mc30, and Md45A. Error bars represent the bootstrap-estimated 95% confidence intervals.

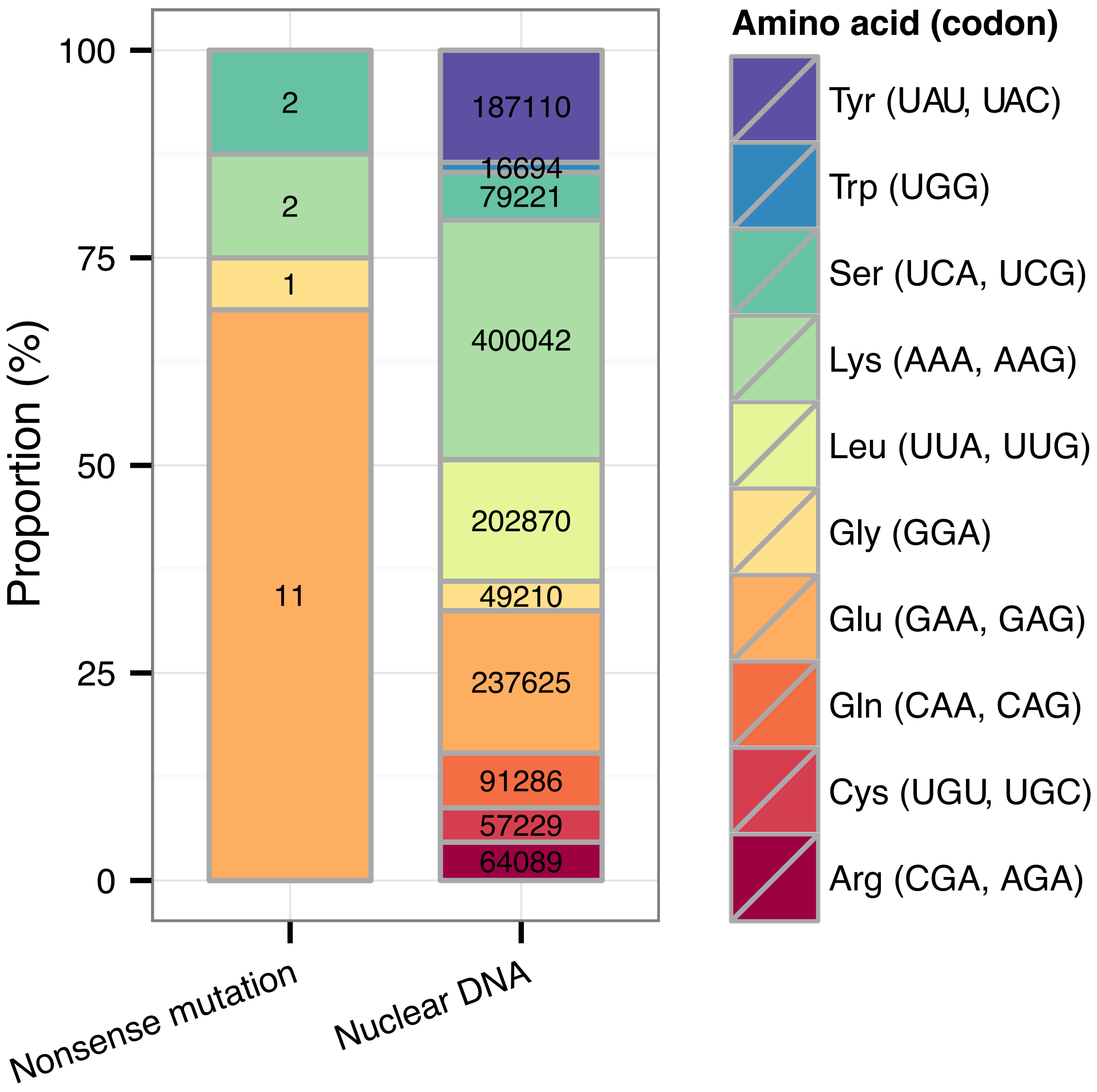


**Supplementary Figure S2. Significantly frequent occurrence of nonsense mutations at Glu codons in PbMut.** The frequencies of codons with nonsense mutations in PbMut and those of possible codons causing nonsense mutation with a single base substitution in the *P. berghei* genome are shown.

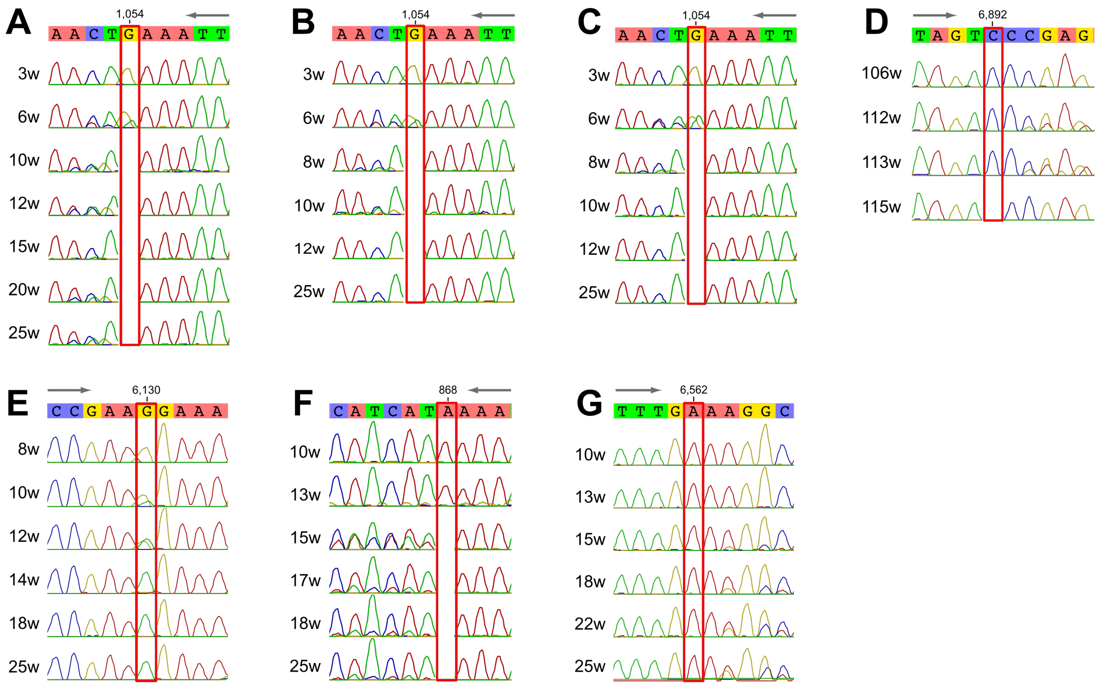


**Supplementary Figure S3. Sanger sequencing of the AP2-G gene (PBANKA_143750) in PbMut and PbWT samples at the various weekly time points (A, Ma; B, Mb; C, Mc; D, Md; E, Wa; F, Wb; and G, Wc).** Arrows indicate the direction of sequencing. Overlapping signals indicate the mixed populations of wild-type and mutant genotypes.

| Organism | Start | Sequence | End |
|---|---|---|---|
| *Plasmodium berghei* ANKA (CDS45138) | 792 | K L E F E K V Y C P Y | 802 |
| *Trypanosoma brucei* (AAQ15638) | 715 | R L E F E K V Y Y P F | 725 |
| *Saccharomyces cerevisiae* (AHY74910) | 798 | N L E F E K A Y F P Y | 808 |
| *Drosophila melanogaster* (AAF49555) | 780 | K L E F E K V Y Y P Y | 790 |
| *Homo sapiens* (AAH08800) | 791 | R L E F E K V Y F P Y | 801 |

**Supplementary Figure S4. Alignment of the amino acid sequences of DNA polymerase δ.** E800 in yeast Pol δ and its corresponding amino acid sites in other organisms are framed by a red rectangle.

**Supplementary Table S1. Summary of sequencing results.**

| Type | PbWT | | | PbMut | | | | | | | | | | | | |
|---|---|---|---|---|---|---|---|---|---|---|---|---|---|---|---|---|
| Sample | Wa29 | Wb28 | Wc29 | Ma28 | Mb29 | Mc30 | Md45A | Md45B | Md83A | Md83B | Md122A | Md122B | Md164 | Ma25P | Mb25P | Mc26P |
| *In vivo* passaged days | 206 | 199 | 203 | 196 | 204 | 209 | 314 | 312 | 576 | 567 | 854 | 855 | 1149 | 174 | 178 | 181 |
| Sequencer used* | HiSeq | HiSeq | HiSeq | HiSeq | HiSeq | HiSeq | HiSeq | HiSeq | GA-IIx | HiSeq | HiSeq | HiSeq | HiSeq | HiSeq | HiSeq | HiSeq |
| Read length (base) | 101 | 101 | 101 | 101 | 101 | 101 | 91 | 91 | 100 | 91 | 91 | 91 | 101 | 101 | 101 | 101 |
| Sequence data used for mapping (Gb) | 1.5 | 1.4 | 1.5 | 1.5 | 1.2 | 1.3 | 1.1 | 1.2 | 1.1 | 1.1 | 1.2 | 1.2 | 1.1 | 5.0 | 4.3 | 4.2 |
| Bases in chromosomes with 10 or more reads (Mb) | 17.3 | 17.6 | 17.4 | 17.6 | 17.0 | 14.6 | 16.9 | 17.0 | 17.8 | 16.1 | 16.7 | 17.2 | 16.7 | 17.7 | 17.9 | 18.0 |
| Total number of base substitutions | 2 | 2 | 0 | 55 | 30 | 44 | 84 | 88 | 137 | 139 | 181 | 180 | 223 | 42 | 32 | 64 |
| Overall base substitution rate ($x10^{-9}$) | 0.55 | 0.57 | 0 | 15.7 | 8.6 | 14.3 | 15.6 | 16.4 | 13.2 | 15.1 | 12.5 | 12.1 | 11.5 | — | — | — |
| Bases in protein-coding regions with 10 or more reads (Mb) | 9.9 | 10.0 | 9.9 | 10.0 | 9.8 | 9.1 | 9.8 | 9.8 | 10.0 | 9.6 | 9.8 | 9.9 | 9.8 | 10.0 | 10.0 | 10.0 |
| Number of synonymous mutations | 0 | 0 | 0 | 5 | 4 | 6 | 3 | 3 | 12 | 9 | 18 | 18 | 20 | 4 | 3 | 7 |
| Number of missense mutations | 0 | 0 | 0 | 29 | 14 | 23 | 54 | 56 | 73 | 79 | 86 | 89 | 118 | 21 | 20 | 32 |
| Number of nonsense mutations | 1 | 0 | 0 | 3 | 0 | 0 | 2 | 3 | 3 | 4 | 4 | 4 | 6 | 1 | 1 | 1 |
| Number of mutations in ribosomal RNA | 0 | 0 | 0 | 0 | 0 | 0 | 0 | 0 | 0 | 0 | 0 | 0 | 0 | 0 | 0 | 1 |
| Bases in introns with 10 or more reads (Mb) | 1.2 | 1.2 | 1.2 | 1.2 | 1.2 | 1.0 | 1.2 | 1.2 | 1.2 | 1.1 | 1.1 | 1.2 | 1.1 | 1.2 | 1.2 | 1.2 |
| Number of substitutions in introns | 1 | 0 | 0 | 3 | 1 | 4 | 2 | 2 | 3 | 2 | 8 | 7 | 11 | 7 | 1 | 6 |
| Bases in intergenic regions with 10 or more reads (Mb) | 6.2 | 6.5 | 6.3 | 6.5 | 6.0 | 4.5 | 5.8 | 5.9 | 6.0 | 6.6 | 5.4 | 5.8 | 6.1 | 6.5 | 6.6 | 6.7 |
| Number of substitutions in intergenic regions | 0 | 2 | 0 | 15 | 11 | 11 | 23 | 24 | 46 | 45 | 65 | 62 | 68 | 9 | 7 | 17 |
| Base substitution rate in intergenic regions ($x10^{-9}$) | 0 | 1.56 | 0 | 11.8 | 8.98 | 11.7 | 12.3 | 12.8 | 12.1 | 14.8 | 13.1 | 11.8 | 10.3 | — | — | — |
| Small indels | 1 | 1 | 2 | 1 | 1 | 3 | 3 | 3 | 4 | 5 | 8 | 9 | 9 | — | — | — |

*GA-IIx, Illumina Genome Analyzer IIx; HiSeq, Illumina HiSeq 2000

**Supplementary Table S3. Indel mutations in detected in this study.**

| Chr | Position | Reference | Alternative | Type of mutation | Region | Effect | Gene ID | Product (total amino acid length) | AA position | Clone |
|---|---|---|---|---|---|---|---|---|---|---|
| berg04 | 446168 | TA | T | Deletion:1 | Intergenic | | | | | Md45A, Md45B, Md83A, Md83B, Md122A, Md122B, Md164 |
| berg06 | 744103 | AT | A | Deletion:1 | Intergenic | | | | | Md164 |
| berg08 | 941898 | G | GT | Insertion:1 | Intergenic | | | | | Md45A, Md45B, Md83A, Md83B, Md122A, Md122B, Md164 |
| berg09 | 245087 | C | CTATT | Insertion:4 | Intergenic | | | | | Wc29 |
| berg09 | 1475602 | GATA | G | Deletion:3 | Exon | Codon deletion | PBANKA_093960 | polyadenylate-binding protein-interacting protein 1, putative (3296) | D2339 | Md122A, Md122B, Md164 |
| berg10 | 643280 | CA | C | Deletion:1 | Intergenic | | | | | Mc30 |
| berg11 | 491606 | C | CAT | Insertion:2 | Intron | | PBANKA_111310 | GTP-binding protein, putative (282) | | Md83A, Md83B, Md122A, Md122B, Md164 |
| berg11 | 860773 | CAT | C | Deletion:2 | Intergenic | | | | | Md122A, Md122B, Md164 |
| berg11 | 1686820 | TA | T | Deletion:1 | Intergenic | | | | | Mc30 |
| berg12 | 1361239 | G | GTGTA | Insertion:4 | Intergenic | | | | | Wa29 |
| berg13 | 30916 | TA | T | Deletion:1 | Intergenic | | | | | Md122A, Md122B, Md164 |
| berg13 | 474156 | T | TA | Insertion:1 | Exon | Frameshift | PBANKA_131090 | conserved Plasmodium protein, unknown function (121) | Y39 | Md83B |
| berg13 | 2414136 | AT | A | Deletion:1 | Exon | Frameshift | PBANKA_136380 | phosphatidylinositol transfer protein, putative (1513) | D505 | Md45A, Md45B, Md83A, Md83B, Md122A, Md122B, Md164 |
| berg14 | 1366415 | TA | T | Deletion:1 | Exon | Frameshift | PBANKA_143750 | transcription factor with AP2 domain(s) (AP2-G) (2339) | K290 | Wb28 |
| berg14 | 1366601 | TG | T | Deletion:1 | Exon | Frameshift | PBANKA_143750 | transcription factor with AP2 domain(s) (AP2-G) (2339) | E352 | Ma28, Mb29, Mc30 |
| berg14 | 1372109 | GA | G | Deletion:1 | Exon | Frameshift | PBANKA_143750 | transcription factor with AP2 domain(s) (AP2-G) (2339) | K2188 | Wc29 |
| berg14 | 1372439 | TC | T | Deletion:1 | Exon | Frameshift | PBANKA_143750 | transcription factor with AP2 domain(s) (AP2-G) (2339) | P2298 | Md122A, Md122B, Md164 |
| berg14 | 1976330 | GTATTTTTCCTAA | G | Deletion:12 | Intergenic | | | | | Md122B |

**Supplementary Table S4. Primers for PCR and sanger sequencing.**

| Chr | Pos | Ref | Alt | Forward primer for PCR | Reverse primer for PCR | Primer for sequencing |
|---|---|---|---|---|---|---|
| berg01 | 173173 | A | T | TGAAGCCATAATACCCATAGACTCCA | GGCTGTATCGAATAATTTGCTTATCCA | Reverse primer |
| berg02 | 29269 | G | T | AAGATTTGGTTATCCGCTGCTTACA | TCTGAACGAACTTCTGAACAGCTTTA | TCTCAACTCTAAGCAATCTACAAAT |
| berg02 | 70382 | T | A | GTTCCGACCAGGTATAAATTAACGACCA | AGGAACTCAAAGTACACCAGGAGCA | AGGGAATTGCGTTGACATTAACTAT |
| berg02 | 637504 | A | T | ACAGTGATATAGCAGTAGCAATAATAGT | TGCCCTGAAACTTAAATACTGCACA | AAACGAGAATTAGCACTGAAACTGT |
| berg03 | 296097 | A | T | ACATTGAGGATGAAGAAGATGATGAAGA | GGATAAGTTTGTCCACAACTTTGCCA | Reverse primer |
| berg04 | 26002 | C | A | TGCATACACAAAGCGTTCAGTCCAT | GTGTTTTGAGGAATTATATACCCGACA | Reverse primer |
| berg04 | 446168 | TA | T | GTGAATTGAATAACATTATATGCGCGTGGT | AATTCTTCATTCCCAGAATAATGCTTAGGT | Forward primer |
| berg05 | 418172 | A | G | AAATTCTACGTTTGTCGCCGTGTTA | AAATGTTGTGATCTCCTTTCGGCAT | Forward primer |
| berg06 | 91450 | A | T | CCTTTACAGTTACCCGAATCCCGAGACA | AACTATGCAAAGTGTTAAGTATGGGGTT | Reverse primer |
| berg06 | 744103 | AT | A | TGCACACATGGGAGTAGGTATGTAAGT | GCTATATTTCGCCATGCTCATTACCCT | Forward primer |
| berg07 | 93172 | C | A | AGTAATTTCCGCTTTCCTCTTCATT | TGGTTTGATTTGCTGTGTATTTCTT | Forward primer |
| berg07 | 632042 | G | T | TGAGAGGGAATAGAGAAGGATAGTT | GTGTACTATAATTTGCACCAAAGGA | Forward primer |
| berg07 | 642250 | C | A | TCCAAGCGTATTTATTACATCATTAGGTGT | AGTTATGATAGCAATCAAAGTAAGCAAAGT | Forward primer |
| berg08 | 941898 | G | GT | ACGGTATATAGTGTGCTGAGTAATAGTTGA | TCGTGTCTTTAAATGATTTGACACTGCTCT | Forward primer |
| berg08 | 1216111 | G | T | TTGAGCTCATTGAGGAAAACCCCGATGT | GGCTTCATATTCCCTTTTTAGCAGTTCT | Forward primer |
| berg09 | 170723 | G | T | TGGAGACGACATTCAACATATCGGGACT | TGTGTGTGTGTATTGCTTATATGGGTGT | Forward primer |
| berg09 | 245087 | C | CTATT | TTTAGTTTCGTTGTATTTAAGGTT | AATATACTAGAGGTTTGTTCCC | TCTTTCATTATCATTTATCATTTGT |
| berg09 | 361587 | G | A | GTTTAGCGAATCTGGGAAGCAAGTA | TGAAAGCGTCGTCAATCATTTCTGA | Forward primer |
| berg09 | 898982 | T | A | ACTGTCATTGCCCATAACCACAATA | CACTGTAATGAAATGCAAGTATGTAGAAGA | Forward primer |
| berg09 | 1475602 | GATA | G | GGTCATAATTGGAGCTTAAGAGGTGA | TAGAATATGAATGCCTCCAACCGTC | Forward primer |
| berg09 | 1512465 | C | A | AGAAGTTCGATAAAGACACAAATAGC | AATACTGATATTCTTGGCCGCTTAG | Forward primer |
| berg10 | 505273 | G | T | TGAACAAGGAAAGATAGAAATGCAATCA | CCATCACGACAAGCCTATGAGGATGAGT | Forward primer |
| berg10 | 643280 | CA | C | TTTAACTTTCCCTAACAAACTATTA | AGTGGATAGAATTAAGATGAACA | Reverse primer or TTAAATTCACACAGTTCCCTTT |
| berg11 | 378323 | G | T | ATATGCAAACCAAACAGCACCAGTA | TTCACCTTTAATTGCGTTGAATCACA | Reverse primer |
| berg11 | 491606 | C | CAT | GCATGATGTAATTCTTCAGCCTAAATCGAA | TCATATTCTTTATCAGGGCCGAACGAGGCA | Forward primer |
| berg11 | 860773 | CAT | C | GATAGCGGTAGTAACCCATTACACA | CTCATGAATTGAGACAACATGAGCA | TGTTAATTATACACCCAACGAAACAGA |
| berg11 | 1047640 | C | G | AAAGAATTGAGAATAACAGGGAAATCA | TTATCTTCTGTATCTGTGCCACTTT | Reverse primer |
| berg11 | 1289022 | G | T | ACGCAATGCACATAAAGACCAATGGTGA | GCCCTTCTAGCTCATTTATGCATTGTTCA | Forward primer |
| berg11 | 1686820 | TA | T | ATTATTCATTCTGGCATCCCTATTT | TTAGCATCCTTATGTAGTATTCCG | ATGTTTATGTATTTGTTTACTTCAA |
| berg11 | 1691473 | T | A | CGTTGCGCCATAGGCCCAATATTAGTTT | CTTCATCCAAAAATCCATAGCCATTCCT | Forward primer |
| berg12 | 605329 | T | A | TATTAGTATATGGTGCGGTTCGATT | ACATTCTCAAACAATTCAACAACTG | Reverse primer |
| berg12 | 1270613 | C | A | ACTATAGCGGACATCCCGTTTTTACGGT | TTCTTCAGTTTATGTTCCCGAAGAAGGT | Forward primer |
| berg12 | 1361239 | G | GTGTA | CATGTGCTTATTTACTTGGGCAATTT | CGCCATTAAGCTAGTTCTCATTGT | Forward primer |
| berg12 | 1415198 | C | A | AGGAGACGCAACTCTTGTAGTACCAACT | TCAACACTATTTCCCAAAGATTTACATGCT | Forward primer |
| berg13 | 30916 | TA | T | GCAAAATTGATTTAGGGGACAATAACCCGT | TCAATTTCAGTGCAAAGTAACTCATGGA | Forward primer |
| berg13 | 441957 | C | A | AAGAGTGTACGCAATTTGGCGCGAATAA | CCTGTGCGCAAGTAATGCTTTTACCCTT | Reverse primer |
| berg13 | 474156 | T | TA | GAAATTAGCAGACACAAATGCTCAAATGGA | GGGTGTACCTACGTAATGCTTATCATGCCA | Forward primer |
| berg13 | 569596 | T | A | AGGCTATACTGCACATTTATATCATAC | TAAATTACGAATACATCCCAGGTGA | Forward primer |
| berg13 | 1750739 | C | T | TTCGTTAGCTGCAACAGTCATATACA | CATTACCTCACAATAGTTCGGCGATT | TTTAGTTGGTTGCTTATCAAT |
| berg13 | 1942165 | T | A | GGCTGTAATCGTTCCACAACCAGAT | GGGAATGGGTCGAAACAAGTCGATG | Forward primer |
| berg13 | 2414136 | AT | A | AAAGGAGATTTGTTTGATGAGATTGAGCCT | ACTGAATTACAAACACATTATTCCTACCCT | Forward primer |
| berg14 | 223106 | C | A | CATGAATAATACAGTCATCCTTCCA | AAGTTGCATATTTGTTTGTGACTTT | Forward primer |
| berg14 | 1056453 | C | T | TGACGAATAGACCAATTATGGAATGACT | ATTCGGTTCAAATACCCACACTCTT | ATAAATAAGATGAAATGCGA |
| berg14 | 1366415 | TA | T | ATGATAGTACAAGCGACAGTAGCCA | TCCATGAACAATGTCCTCTCGTTGA | TTTCCCATTTGTGTATTTCCTTTAT |
| berg14 | 1366601 | TG | T | ATGATAGTACAAGCGACAGTAGCCA | TCCATGAACAATGTCCTCTCGTTGA | TTTCCCATTTGTGTATTTCCTTTAT |
| berg14 | 1371678 | G | T | ATGATAGTACAAGCGACAGTAGCCA | TCCATGAACAATGTCCTCTCGTTGA | TTTCCCATTTGTGTATTTCCTTTAT |
| berg14 | 1372109 | GA | G | ATGATAGTACAAGCGACAGTAGCCA | TCCATGAACAATGTCCTCTCGTTGA | TATGAAAAGAGATAATATGGATAAA |
| berg14 | 1372439 | TC | T | GCGCTTTACGATTAGCCGTTGCAATGAA | CGCTAGTTTCGTCATCGCTGCTATTTGT | Forward primer |
| berg14 | 1865484 | T | A | ATTGCTGCGGACAAGTTCGATTCTG | TTTCGCTTCCTCCGGGACCTTAAC | Forward primer |
| berg14 | 1865561 | T | A | ATTGCTGCGGACAAGTTCGATTCTG | TTTCGCTTCCTCCGGGACCTTAAC | Forward primer |
| berg14 | 1976330 | GTATTTTTCCTAA | G | ATTTCTAGAGAGCAGAATGGGTATTTATTT | ATGCGAACAAGTAAGTGGCATTTGA | TGGTGTATAGTTTAGCATATAGGAA |
| berg14 | 2408508 | G | T | ATCAACCTTTCCCTGTTATGGCATC | TCCAGTTTCACTACTATCGGAGGAT | Reverse primer |
